# The Genetic Basis of Variation in Clean Lineages of *Saccharomyces cerevisiae* in Response to Stresses Encountered during Bioethanol Fermentations

**DOI:** 10.1371/journal.pone.0103233

**Published:** 2014-08-12

**Authors:** Darren Greetham, Tithira T. Wimalasena, Kay Leung, Marcus E. Marvin, Yogeshwar Chandelia, Andrew J. Hart, Trevor G. Phister, Gregory A. Tucker, Edward J. Louis, Katherine A. Smart

**Affiliations:** 1 School of Biosciences, University of Nottingham, Sutton Bonington campus, Loughborough, United Kingdom; 2 Centre for Genetic Architecture of Complex Traits, Department of Genetics, University of Leicester, Adrian Building, Leicester, Leicestershire, United Kingdom; University of Nottingham, United Kingdom

## Abstract

*Saccharomyces cerevisiae* is the micro-organism of choice for the conversion of monomeric sugars into bioethanol. Industrial bioethanol fermentations are intrinsically stressful environments for yeast and the adaptive protective response varies between strain backgrounds. With the aim of identifying quantitative trait loci (QTL's) that regulate phenotypic variation, linkage analysis on six F1 crosses from four highly divergent clean lineages of *S. cerevisiae* was performed. Segregants from each cross were assessed for tolerance to a range of stresses encountered during industrial bioethanol fermentations. Tolerance levels within populations of F1 segregants to stress conditions differed and displayed transgressive variation. Linkage analysis resulted in the identification of QTL's for tolerance to weak acid and osmotic stress. We tested candidate genes within loci identified by QTL using reciprocal hemizygosity analysis to ascertain their contribution to the observed phenotypic variation; this approach validated a gene (*COX20*) for weak acid stress and a gene (*RCK2*) for osmotic stress. Hemizygous transformants with a sensitive phenotype carried a *COX20* allele from a weak acid sensitive parent with an alteration in its protein coding compared with other *S. cerevisiae* strains. *RCK2* alleles reveal peptide differences between parental strains and the importance of these changes is currently being ascertained.

## Introduction

Fossil-based hydrocarbon fuels for generating energy, such as coal and crude oil, are not infinite resources and at the present rate of human consumption are predicted to be completely depleted by 2050 [1]. In order to sustain and satisfy the appetite of the planet's developed economies and the increasing demands of newly-emerging industrial nations, alternative ‘renewable’ forms of energy need to be utilised to ease the current rate of fossil fuel consumption and to eventually replace them completely. One such renewable source for these alternative forms of energy is lignocellulosic residue from agricultural, forestry, municipal or industrial processes [2]. Sugars can be released from the lignocellulosic feedstocks using industrial pre-treatment processes, followed by enzymatic digestion and then converted to transportation biofuels, such as bioethanol, biobutanol or biodiesel by microbial fermentation [3]. In order to replace fossil fuels, industrial scale biofuel production from lignocellulose, will rely on the efficient conversion of all the sugars present in the feed stocks to maximise profits, economic viability and importantly, to obtain a smaller carbon footprint.

*Saccharomyces cerevisiae* is currently used for the production of bioethanol. First generation bioethanol production has involved the conversation of hexose sugars present in cash crops such as sugar cane in Brazil and Maize in the United States of America [4]. Future 2^nd^ generation production will rely not only on fermentation of hexose sugars, but also of pentose sugars present in plant cell walls in approximate equal amounts [3]. *S. cerevisiae* cannot currently convert pentose sugars to bioethanol effectively, but studies towards alleviating this problem are underway [5]. To further increase the efficiency of fermentation, the problem of pre-treatment generated inhibitor compounds, and fermentation stresses, also has to be addressed. Pre-treatment of lignocellulose to release constituent sugars results in the formation of aromatic and acidic compounds such as acetic acid, formic acid, furfural, hydroxy-methyl furfural (HMF), levulinic acid and vanillin [6] that are detrimental to the growth of *S. cerevisiae*. In addition, fermentations carried out within bioreactors generate additional difficulties, such as osmotic stress due to high sugar levels, elevated heat and increasing ethanol concentrations [7]–[9]. Thus, resistance to all these fermentation stresses are desirable phenotypic attributes for improved bioethanol productivity.

Five clean lineages (West African, Wine European, Sake, North American and Malaysian) of *S. cerevisiae* represent major clades [10] and have been engineered to enable genetic tractability [11]. When two of these clean lineages are crossed and the resulting F1 hybrids sporulated to generate an F1 offspring population, the progeny display a wide range of phenotypes including transgressive variation [12]. All F1 segregants from six pairwise crosses of four of these clean lineages (West African, Wine European, Sake and North American) have been extensively genotyped and phenotyped for growth in many environmental conditions of ecological relevance [10]. This has enabled these clean lineages to be used as powerful tools and models to determine multigenic traits using QTL analysis. Using these F1 segregants, we have performed phenotypic analysis of metabolic output in the presences of stresses encountered during fermentation of lignocellulosic biomass and determined QTLs governing complex traits important for bioethanol production. By coupling our analysis to selective breeding and evolutionary engineering, novel yeast strains can be produced with inherent properties for improving industrial 2^nd^ generation bioethanol production [13], [14].

## Materials and Methods

### Yeast strains and growth conditions

We selected four representative clean lineage strains (North American (NA): YPS128, West African (WA): DBVPG6044, Sake (SA): Y12, Wine/European (WE): DBVPG6765) [10]. Previously derived stable haploid versions (*ho::HygMX, ura3::KanMX*) from the original wild-type homothallic strains were used [11]. Haploid strains with opposite mating types (*MatA* and *Matα*) were crossed to produce diploid hybrids of the parental isolates. All segregants are available at the National Collection of Yeast Cultures (http://www.ncyc.co.uk/index.html). We used isogenic yeast strain CC26 as the diploid parent of DBVPG6044×Y12 and CC16 as the diploid parent of YPS128×Y12 [11] as the basis for reciprocal heterozygosity and qPCR experiments. BY4741 under non-stress conditions was used as a negative control for qPCR experiments.

For general vegetative growth, either yeast extract peptone dextrose (YPD) medium [1% yeast extract (Oxoid); 2% (w/v) Bacto-peptone (Oxoid); 2% (w/v); 2% (w/v) glucose], or synthetically defined (SD) medium [0.67% (w/v) yeast nitrogen base (YNB) with amino acids and ammonium sulphate; 6% (w/v) glucose] were used. Cultures were cryopreserved in 20% (v/v) glycerol at −80°C.

### Phenotypic microarray analysis

For phenotypic microarray (PM) analysis, medium was prepared using 0.67% (w/v) yeast nitrogen base (YNB) supplemented with 6% (w/v) glucose, 2.6 µl of yeast nutrient supplement mixture (NS×48- 24 mM adenine-HCl, 4.8 mM L-histidine HCl monohydrate, 48 mM L-leucine, 24 mM L-lysine-HCl, 12 mM L-methionine, 12 mM L-tryptophan and 14.4 mM uracil) and 0.2 µl of dye D (Biolog, Hayward, CA, USA). The final volume was made up to 30 µL using sterile distilled water, inhibitory compounds were added as appropriate and water removed to maintain a 30 µL volume. Stock solutions (1 M) of the aliphatic weak acids acetic acid, formic and levulinic acid were prepared using reverse osmosis (RO) sterilised water; furfural, HMF and vanillin were prepared as 1 M stock solutions in 100% ethanol. A stock solution of 80% sorbitol (w/v) was prepared and adjusted to generate 10% and 15% (w/v) concentrations in a final volume of 120 µl. For ethanol 10% (v/v) and 15% (v/v) was used to induce ethanol stress. Temperature was adjusted to either 30°C, 35°C, or 40°C and data was taken at 15 min intervals for 96 hours at 30°C and 35°C, and for 24 hours at 40°C. Assays at 40°C were limited in terms of time due to the effect of evaporation if measured for 96 hours. Medium containing glucose, YNB, NS, dye, water and inhibitory compounds (as appropriate) were prepared in bulk corresponding to the number of wells for that particular experiment and 30 µL aliquoted out per well as appropriate.

Strains were prepared for inoculation onto PM assay plates as follows. Glycerol stocks stored at −80°C were streaked on to YPD plates to obtain single colonies and incubated at 30°C for approximately 48 hrs. Two to three colonies from each strain were then patched on a fresh YPD plate and incubated overnight at 30°C. Cells were then inoculated into sterile water in 20×100 mm test tubes and adjusted to a transmittance of 62% (∼5×10^6^ cells.mL^−1^) using sterile distilled water and a turbidometer. Cell suspensions for the inoculum were then prepared by mixing 125 µl of these cells and 2.5 mL of IFY buffer (Biolog, USA) and the final volume adjusted to 3 mL using RO sterile distilled water, 90 µl of this mix was inoculated to each well in a Biolog 96-well plate. Anaerobic conditions were generated by placing each plate into a PM gas bag (Biolog, Hayward, CA, USA) and vacuum packed using an Audion VMS43 vacuum chamber (Audion Elektro BV, Netherlands).

An OmniLog reader (Biolog, Hayward, CA, USA) was used to photograph the plates at 15 min intervals to measure dye conversion, the pixel intensity in each well was then converted to a signal value reflecting cell metabolic output. After completion of each run, the signal data was exported from the Biolog software and analysed using Microsoft Excel. In all cases, a minimum of three replicate PM assay runs were conducted, and the mean signal values are presented. Percentage redox signal intensity was calculated using the redox signal intensity values at 48 hrs for each stress condition and normalised by dividing this value by the value under non-stress conditions at the same time point except for thermal stress at 40°C, where this was calculated using the redox signal intensity values at 24 hours for control and stressed conditions.

### R statistical computing environment

Data from the 48 hr time points were analysed using Linkage analysis was performed with jQTL (http://churchill.jax.org/software/jqtl.shtml), a java graphical interface for R/qtl package x86_64-w64-mingw32/x64 [15], data converted into comma delimited files and run on a R workspace. RGui 64 bit is a free to use software for statistical analysis package http://cran.r-project.org/bin/windows/base/. This package was used to compare sugar utilisation of haploid *S. cerevisiae* yeast strains.

### Linkage Analysis

Linkage analysis was performed with the jQTL software (Churchill group) [16]; we calculated logarithm of the odds (LOD) scores using the nonparametric model. The significance of a QTL was determined from permutations. For each trait and cross, we permutated the phenotype values within tetrads 1000 times, recording the maximum LOD score each time. We called a QTL significant if its LOD score was greater than the 0.05 tail of the 1000 permuted LOD scores.

### Reciprocal Hemizygosity Analysis

To validate the presence of contributing genes within QTL's, we used a modified reciprocal hemizygosity assay [17]. The *URA3* gene (essential for pyrimidine biosynthesis) previously deleted in parental strains [11] was used as an auxotrophic selectable marker.

Reciprocal hemizygosity analysis was performed for genes lying within QTL's identified on chromosomes IV and XIII (acetic acid tolerance) and chromosome XII (osmotic stress tolerance). Using crosses of parental strains (CC16: YPS128×Y12, and CC26: Y12×DBVPG6044) each allele of each gene was deleted, resulting in a hemizygous diploid carrying one parental allele [17]. To generate gene deletions, synthetic oligonucleotide primers were designed to produce disruption cassettes. Each primer contained 80-bp of sequence homology for the selected gene's open reading frame (ORF) immediately flanking the start and stop codons (Table 1). The addition of sequence homologous to pAG60 (Euroscarf Germany) at the 3′ end of each primer allowed the amplification of the *Kluyveromyces lactis URA3* gene as an auxotrophic selectable marker. The *URA3* gene from *Kluyveromyces lactis*, *KlURA3*, functions in *S. cerevisiae* but has little sequence homology which prevents recombination with the native *ScURA3* gene locus to improve transformation efficiency. Amplification by PCR results in *KlURA3* flanked by 80-bp of sequence homologous to the target gene to be deleted. PCR amplified *URA*3 deletion cassettes targeting each gene were transformed into each corresponding heterozygote hybrid diploid parent using methods described in Gietz and Schiestl, 2007 [18]. Positive transformants were selected on SD agar plates supplemented with all amino acids supplements, minus uracil (–URA) and incubated at 30°C until colonies were formed. Single transformants were picked and re-streaked onto fresh selective plates to ensure pure isolates. Single colonies from these plates were patched and used for further analysis.

**Table 1:** Primers utilised during this study.

Gene/Application	Primer Sequence 5′ to 3′
*COX20* deletion forward primer	AAACTCCACTGCTCGGTAAAGCATTGTAGTGAAGTCCACAGCAGTGCGTAACGAGCAGCTCAACAGTTAATATAAAGATGagcttttcaattcaattcatcat
*COX20* deletion reverse primer	TTTCGGAGAAATGTTGCATATATACATAGGAAAACGGTTAAAAGGCCCTGCTTCTACCTTCTGTTTCCCCCTCGTTCTTTagctttttctttccaatt
*RCK20* deletion forward primer	ACATTTAACGATTGGAAAAGACGAAAGTATTGTTAAGAGTACTGCTTATTTAGAGAGGATCAAACAAAATCTCTTCGagcttttcaattcaattcatcat
*RCK20* deletion reverse primer	TATACTTGTAGAAGGAGTTTAATGTATATATATCTTTTAAAAAGGAATAGGTAAAAAGATTGAAACAGAAGGGAAAGTTGagctttttctttccaatt
*COX20*F sequencing forward primer	GAAACGCGAGCTGAGAAGGG
*CPX20* R sequencing reverse primer	CGGCATGCAAGACCAGTCAA
*RCK2F* sequencing forward primer	AGAAAAGACGGATCGGCCAA
*RCK2R* sequencing reverse primer	GGAAGGGGCGAACAATG

Sequences in lower case indicates target site corresponding to *URA3* in the pAG60 cassette.

### Sequence analysis

To confirm allelic variation in strains during reciprocal hemizygosity analysis sequencing was used. PCR amplification was performed using primers (COX20F and COX20R: RCK2F and RCK2R Table 1) with an initial denaturation of 98°C for 30 s followed by 35 cycles of 98°C for 10S; 60°C for 30S, 72°C for 2 min and a final elongation for 72°C for 5 min using Phusion Taq polymerase (NEB, Ipswich, UK). PCR generated amplicons were purified using commercially available purification columns (Qiagen, Netherlands) and sequenced using the MWG Eurofin service (Ebersberg, Germany). Six tranformants were sequenced for each gene.

Each sequence read from the amplified PCR products were compared against sequences from the *Saccharomyces* genome resequencing project available on the Welcome Trust Sanger Institutes website (https://www.sanger.ac.uk/research/projects/genomeinformatics/sgrp.html) using Vector NTI Advance version11 (Invitrogen, Paisley, UK). Amino acid sequence differences were identified in Cox20p and Rck2p proteins from each clean lineage using the BLAST tool in the SGRP site

### Quantative PCR analysis

The diploid hybrid strains used to generate the reciprocal hemizygotes were used in qPCR analysis, (CC26-for osmotic stress and CC16 for acetic acid stress). These were grown to the mid-logarithmic stage of growth in YPD at 30°C and stressed by the addition of 25 mM acetic acid, or 20% sorbitol for 15 min, rotated at 150 rpm. Cells were broken with glass beads using a MagNalyser (Roche, Burges Hill, UK) bead beater for 30 seconds at 4°C, before incubating on ice for 15 min to precipitate proteins. Cell debris and proteins were harvested by centrifugation for 15 min (17,000× g at 4°C). The cell-free supernatant was used for the extraction of total RNA using an isolation kit from Qiagen (Hilden, Germany) and cDNA prepared using a first strand cDNA synthesis kit (GE Healthcare, Bucks, UK). Transcriptional levels were determined by qPCR using the following conditions follows: 0.5 ng/µl cDNA, 6.25 µM forward primer, 6.25 µM reverse primer, 5 µl of 2× SYBR Green master mix (Applied Bio Systems) and made up to 20 µl using molecular grade water. All data was compared against *ACT1* as an internal normaliser and expression data from genes within the relevant loci were presented as fold-change in comparison to *ACT1* transcript levels in control and stress conditions.

## Results

### The phenotypic response of haploid F1 segregants derived from a six pairwise crosses to stresses encountered during bioethanol fermentation

**Figure 1:**
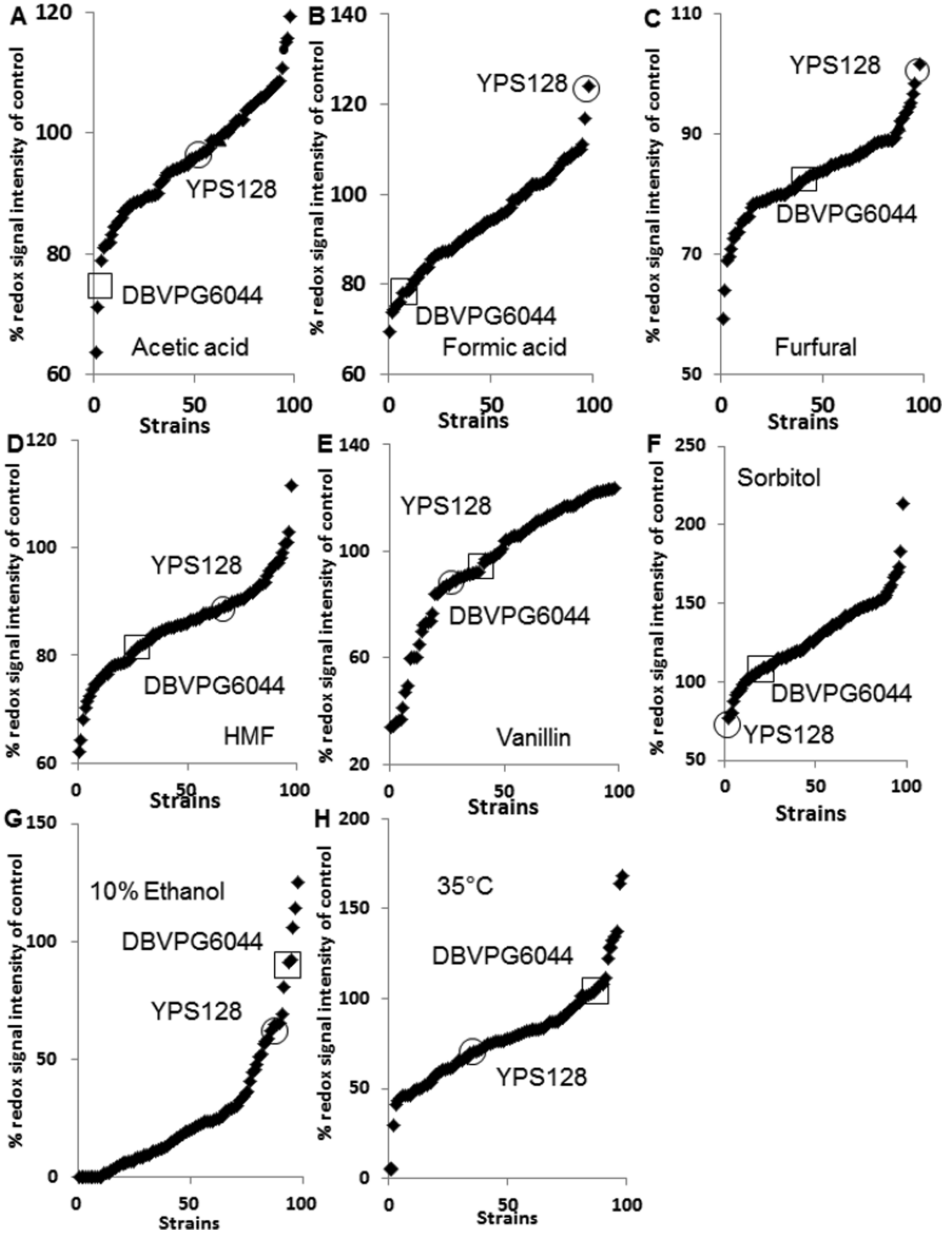
Phenotypic microarray analysis (redox signal intensity) of F1 haploid segregants from a Y12×DBVPG6044 cross. Tolerance to (A) 10% and 15% sorbitol, (B) 10% and 15% ethanol, (C) 35°C and 40°C, (D) 25 mM acetic acid, (E) 10 mM formic acid, (F) 10 mM levulinic acid, (G) 5 mM HMF, (H) 5 mM furfural and (I) 5 mM vanillin are shown. The Y axis represents the % of RSI (redox signal intensity) where wells containing the listed stresses are compared to unstressed conditions. All yeast cells were grown in minimal medium with 6% glucose added at 30°C with the final data shown at the 25 hr time point. The values shown are an average of triplicate experiments including standard deviations.

Using a phenotypic microarray assay, we analysed 96 haploid F1 segregants, derived from six pairwise crosses between four clean lineage strains of *S. cerevisiae*, for their response to stresses encountered during bioethanol fermentation. By comparing profiles of stressed cells to non-stressed control cells, (defined here as the percentage of redox signal intensity to that of a control) we determined the response of each F1 segregant population to each individual stress from each cross. Typical results from one of these crosses are shown in Figure 1 A–H (96 haploid F1 segregants plus parental strains) and for other crosses as Figure S1 and data S1, S2, S3, S4, S5, S6. These plots demonstrated considerable phenotypic variation and which was observed in all populations of haploid segregants and to every stress assayed, and did not correlate with the phenotypic response of either parental strain (Figures 1A–1H). This observation of continuous variation among offspring with no large step changes is consistent with being polygenic for each individual stress.

### Transgressive variation with some better than either parent in the segregant populations is not universal

**Figure 2:**
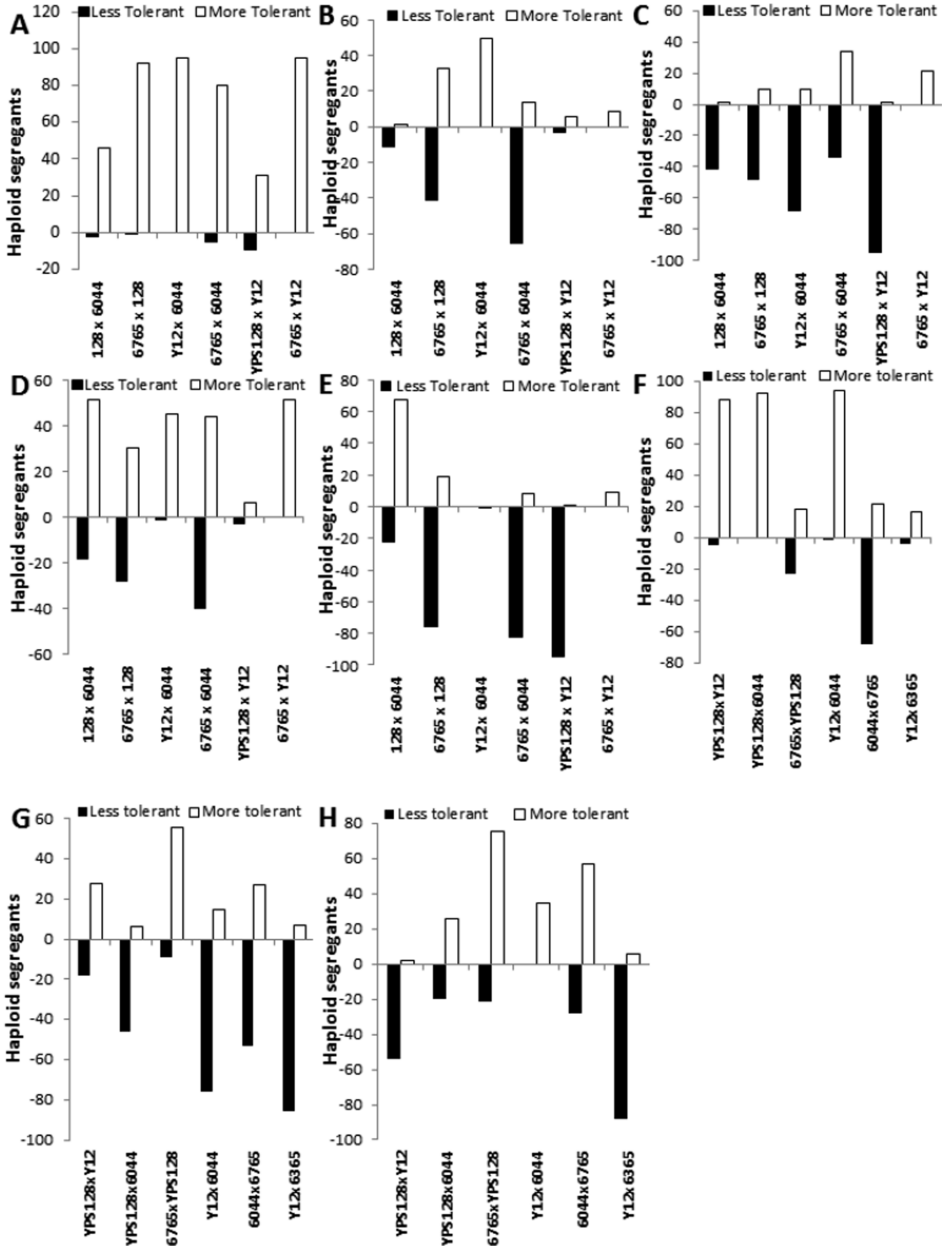
Assessment of variation of yeast populations to stresses encountered during bioethanol fermentations. F1 segregants from six pairwise crosses of four parental *S. cerevisiae* clean lineages were tested for (A) acetic acid, (B) formic acid, (C) HMF, (D) furfural (E) vanillin (F) sorbitol, (G) ethanol and (H) thermal (35°C) stress. Each population exhibited a range of tolerance and sensitivity beyond the parameters set by the phenotypic response of either parent.

The local neutrality hypothesis has been defined as the process of shaping the yeast genotype-phenotype map causing large differences in fitness within a population [19]. This hypothesis suggests that loss-of function mutations in parental lineages promote a strong bias towards superior F1 hybrids compares to parental yeast strains, however, how F1 haploid segregants perform is more complex as they will contain multiple bad combinations revealed in their haploid status. We characterised the phenotypic response of each of the populations of F1 haploid segregants, as compared to their parents for tolerance to a range of stress conditions (Figure 2). For stress conditions such as acetic acid, or HMF, there was a clear improvement in the performance of the offspring when compared to their parents (Figures 2A and 2E). Response to formic acid, sorbitol and temperature stress was dependent on the particular population screened. In some populations, an increase in tolerance, when compared with either parent was observed e.g. Y12×DBVPG6044 to formic acid; (Figure 2B), other populations displayed sensitivity to the same stress (DBVPG6044×DBVPG6765 to formic acid) (Figure 2B). However, for population responses to furfural, vanillin and ethanol there was a reduction in tolerance in the F1 progeny when compared with their parental strains (Figures 2C, 2E and 2G). However, even when in general performances of the F1 haploids were worse than either parent, we still observed individuals within the population which outperformed either parent.

### Population response to one stress can be linked to tolerance to other stresses

**Figure 3:**
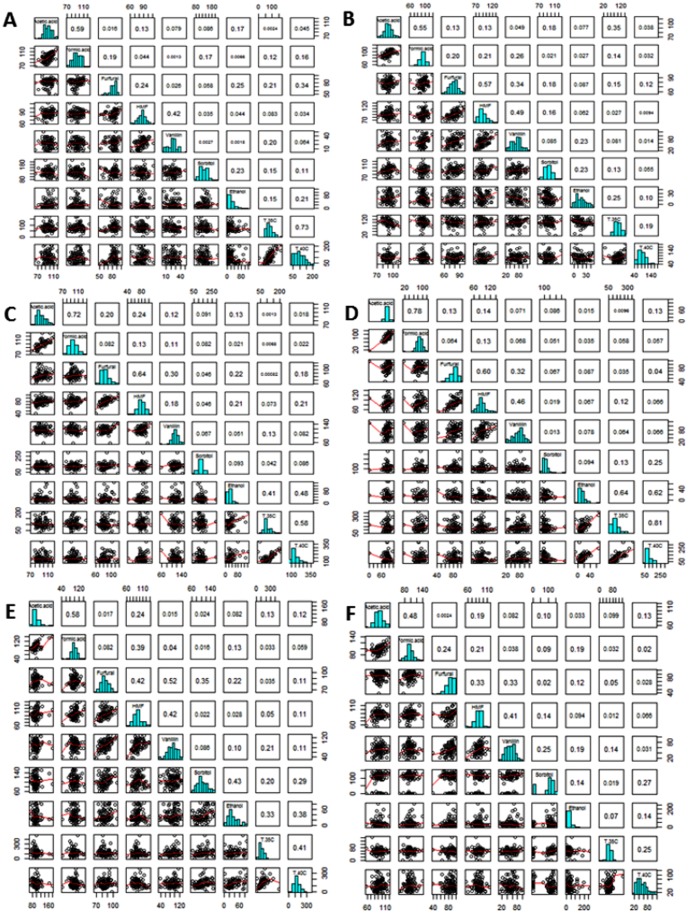
Statistical comparison (using R) of F1 haploid segregants. F1 haploid segregants were grown under stress conditions from crosses (A) Y12×YPS128, (B) YPS128×DBVPG6765, (C) DBVPG6044×DBVPG6765, (D) Y12×DBVPG6044, (E) DBVPG6765×Y12 and (F) YPS128×DBVPG6044 for shared phenotypic response to acetic acid, formic acid, furfural, HMF, vanillin, osmotic (sorbitol), ethanol and temperature (35°C and 40°C).

Ranking of F1 haploid segregants according to their response to an individual stress allowed us to look for shared phenotypes with respect to their individual responses to the other stress inducing conditions. Using this approach it was observed that the haploid segregants response to acetic acid tended to correlate with their response to formic acid (Figures 3A–3F). We also observed that haploid segregants populations stressed with HMF, furfural and vanillin also shared common phenotypic responses (Figures 3A–3F). However, there were exceptions to this observation, as there was little correlation in response to furfural and vanillin in the F1 population derived from the Y12×YPS128 cross (Figure 3A), the same was observed between HMF and vanillin stress in haploids segregants derived from a DBVPG6044×DBVPG6765 cross (Figure 3C). There was also an association in the phenotypic response to osmotic stress (sorbitol) and ethanol stress in some F1 segregant haploid populations such as DBVPG6765×Y12 (Figure 3E) but not in others such as the DBVPG6044×DBVPG6765 cross (Figure 3C). In general, data from temperature stressed F1 segregant haploid populations correlated well (Figures 3A, 3C, 3D, and 3E). However, some populations failed to show this correlation (Figures 3B and 3F).

### Identification of QTL's for stresses encountered in bioethanol fermentation

Inhibitory compounds released during pre-treatment processes affect microbial growth and therefore the efficiency of bioethanol production. Using QTL analysis, we identified three loci which to a degree overlapped each other on chromosome IV under acetic acid stress and formic acid stress, from different crosses (Table 2). A further locus was identified on chromosome XIII from the Y12×DBVPG6044 cross for acetic acid tolerance; this cross also generated a locus in response to formic acid on chromosome XI (Table 2). Additional loci were identified on chromosome XII for tolerance to osmotic stress under anaerobic conditions from the YPS128×DBVPG6765 and YPS128×Y12 crosses (Table 2).

**Table 2:** Linkage analysis for acetic acid and osmotic stress from different segregant populations.

Stress/growth conditions	Cross	Chromosome	QTL
Acetic acid aerobic Acetic acid anaerobic	Y12×DBVPG6044 Y12×DBVPG6044 Y12×DBVPG6044 Y12×DBVPG6044 DBVPG6044×DBVPG6765	IV XIII IV XIII XIII	921–1021 351–451 925–1025 304–405 801–901
Formic acid anaerobic	YPS128×DBVPG6765 DBVPG6044×DBVPG6765	IV XIII XI	935–1035 205–305 7–107
Osmotic stress anaerobic	YPS128×DBVPG6765 YPS128×DBVPG6765 YPS128×Y12	III XII XII	51–151 599–699 389–489

### Identifying genes present in QTLs involved in yeast response to stress

All genes present within the identified QTLs are listed (data S7), as each QTL contained between 40 and 60 genes and we focused our research on the QTL's identified on chromosomes IV (acetic acid tolerance) and XII (osmotic tolerance), as they were identified from different populations and growth conditions.

**Figure 4:**
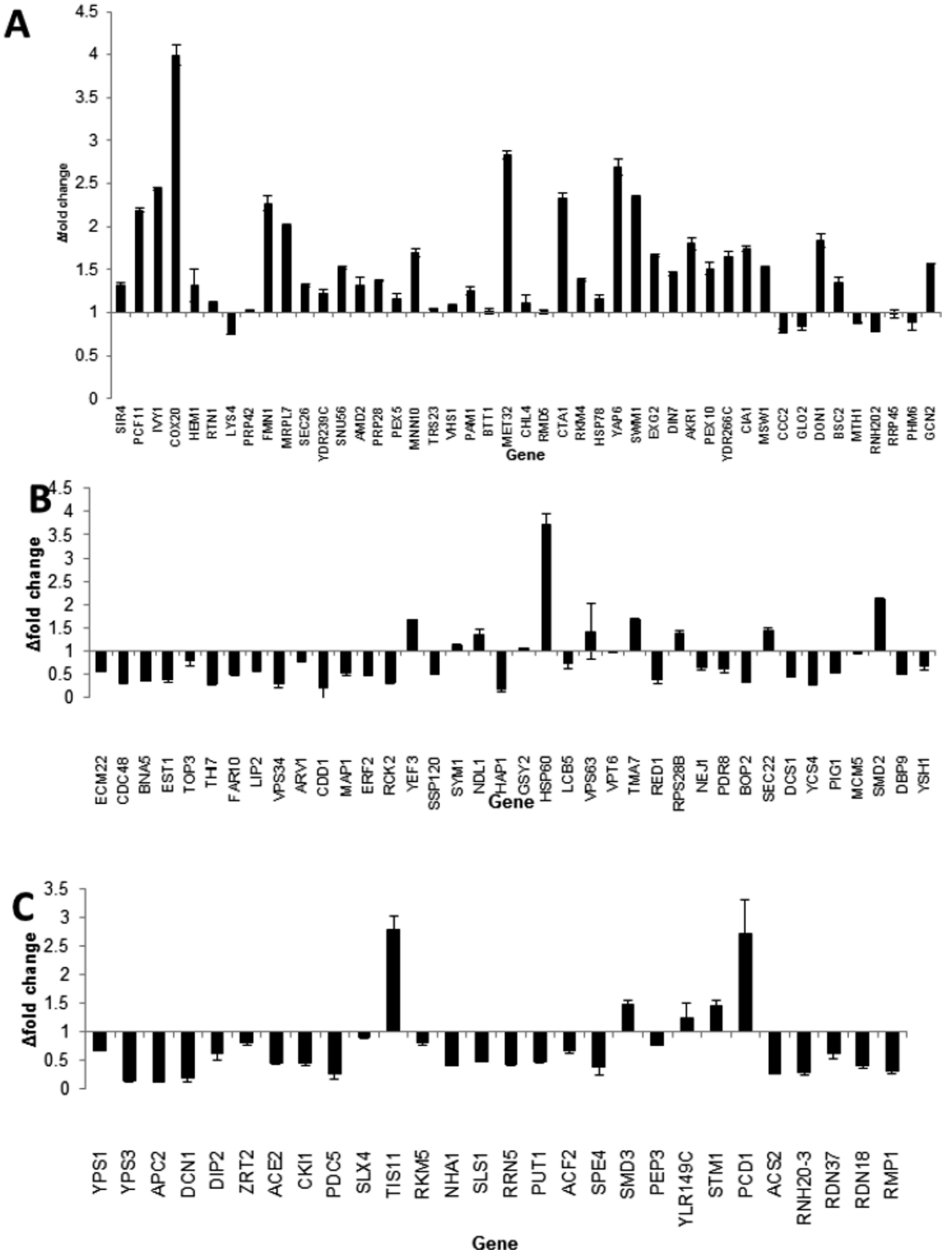
Expression data for genes using qPCR under (A) acetic acid stress present in loci identified on chromosome IV (region 921–1021) in the isogenic diploid parental strain Y12×DBVPG6044, (B) Expression data for genes present in loci identified on chromosome XII (region 599–699 kb) under osmotic stress in the isogenic diploid parental YPS128×DBVPH6765 and (C) Expression data for genes present in loci identified on chromosome XII (region 389–489 kb) under osmotic stress in the isogenic diploid parental YPS128×Y12.

Expression data from the loci identified under acetic acid stress on chromosome IV in the hybrid DBVPG6044×Y12 (Figure 4), identified genes up-regulated such as mitochondrial cytochrome C oxidase assembly gene *COX20*, (Figure 4A). Cytochrome C oxidase activity has been associated with acetic acid induced programmed cell death [20]. Furthermore, expression data from all the genes within the loci identified under osmotic stress on chromosome XII in the hybrid (YPS128×DBVPG6765), identified genes up-regulated under osmotic stress including Hsp60p a known heat shock protein in *S. cerevisiae* [21], [22], It was observed that the majority of the genes present in this locus (area corresponding to 599–699 kb) were down-regulated under osmotic stress (Figure 4B). This was similar for the expression data for the genes present in the locus (area corresponding to 389–489 kb) identified from the YPS128 and Y12 cross, five genes were up-regulated *TIS11*, *SMD3*, *STM1*, *YLR149c* and *PCD1* (Figure 4C). Amongst those genes down-regulated is *PUT1* which has been identified as important in yeast as a response to osmotic stress [23].

### Dissection of weak acid QTL's from chromosomes IV and XIII

**Figure 5:**
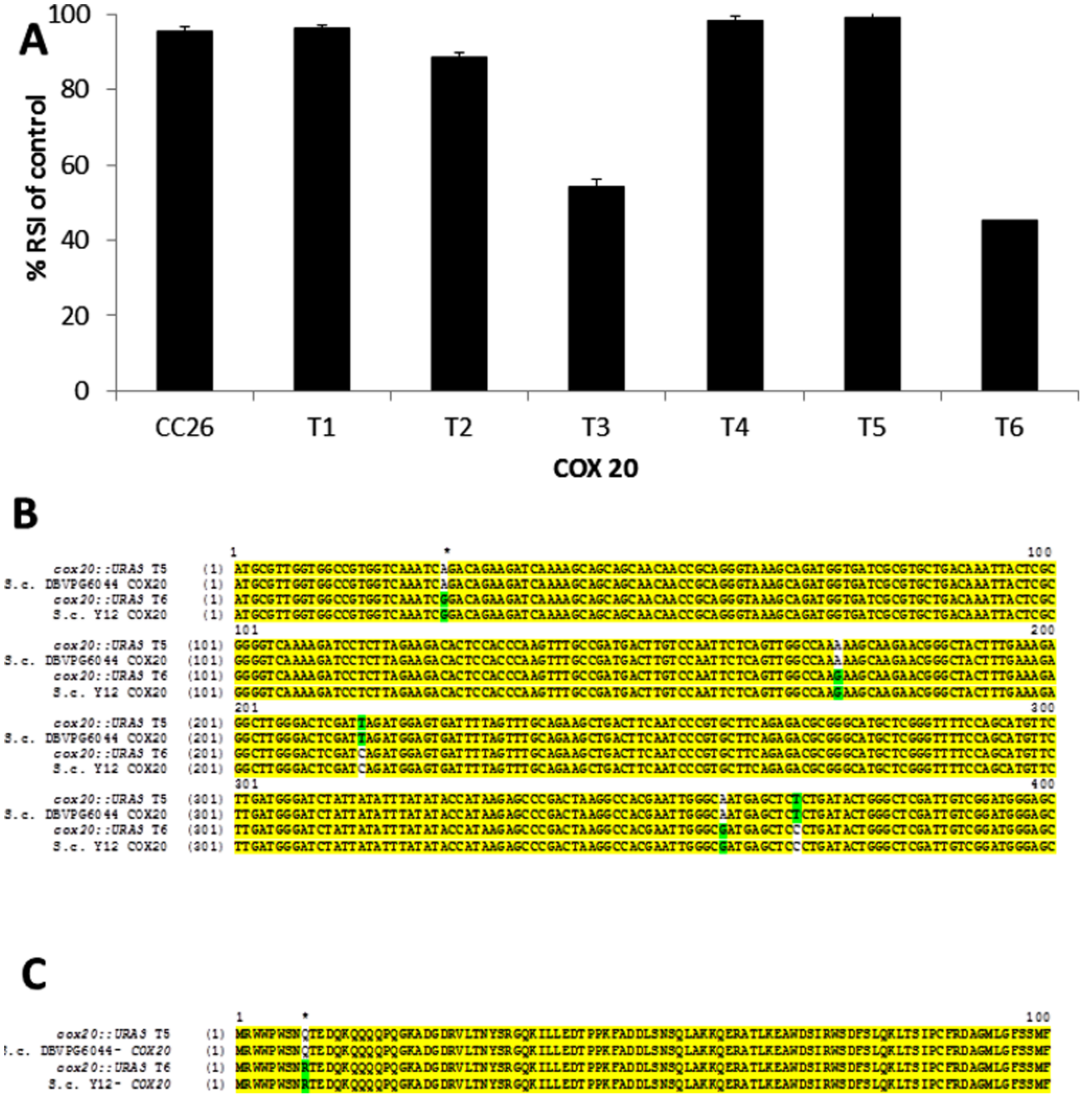
Phenotypic microarray screening of (A) heterozygous diploid transformants (transformants labelled T1–T6) harbouring reciprocal deletions of COX20 alleles to acetic acid stress. DNA sequence comparisons (B) and protein sequence comparisons (C) of T5 and T6 transformants are shown along with their parental strains DBVPG6044 and Y12.

Using the expression data under acetic acid stress and the putative roles of genes underlying the two QTL, following a review of gene function, a number of candidate genes were selected for further testing. These were *ADH3*, *GCN2*, *MSN2*, *COX20* and *AAC1*. All of these candidates were subjected to reciprocal hemizygosity analysis. A distinct segregation into tolerant and sensitive heterozygous diploid transformants was observed in the case of *COX20*; sequencing of the remaining *COX20* allele in each case revealed that sensitive transformants carried the *COX20* allele inherited from *S. cerevisiae* strain Y12. We had previously shown that Y12 displays sensitivity to acetic acid when compared with other *Saccharomyces spp* strains, whereas, DBVPG6044 is more tolerant [24]. Sequence comparison of alleles from both parents revealed that the *COX20* gene of Y12 harboured a glutamic acid to arginine change at position 9 (Figure 5C). However, glutamic acid is the most frequent residue at this position in the *COX20* gene within the *Saccharomyces* spp (data S8). Within *S. cerevisiae* and *S. paradoxus*, only *S. cerevisiae* strains isolated from sake fermentations (K11, Y9 and Y12) contained an arginine residue at position 9 (data S8). Analysis of *COX20* genes from other *Saccharomyces* spp yeast revealed that none contained an arginine residue at position 9 in their predicted *COX20* peptides (data S8). Reciprocal hemizygosity analysis of the other candidate genes tested failed to show any observable variation checked by performance using the phenotypic arrays and sequencing alleles from the resultant transformants (data not shown).

### Dissection of the osmotic QTL from chromosome XII

Expression data from the loci identified on chromosome XII from the YPS128×DBVPG6765 cross highlighted genes which were up-regulated under osmotic stress including Hsp60p, a known heat shock protein in *S. cerevisiae* [21], [22], It was observed that the majority of the genes were down-regulated under osmotic stress (Figure 4B), however, a few genes were significantly up-regulated such as *HSP60*, *TIS11*, and *PCD1* (Figures 4B and 4C). Amongst genes down-regulated is *PUT1* which has been identified as important in yeast as a response to osmotic stress [23] (Figure 4C).

**Figure 6:**
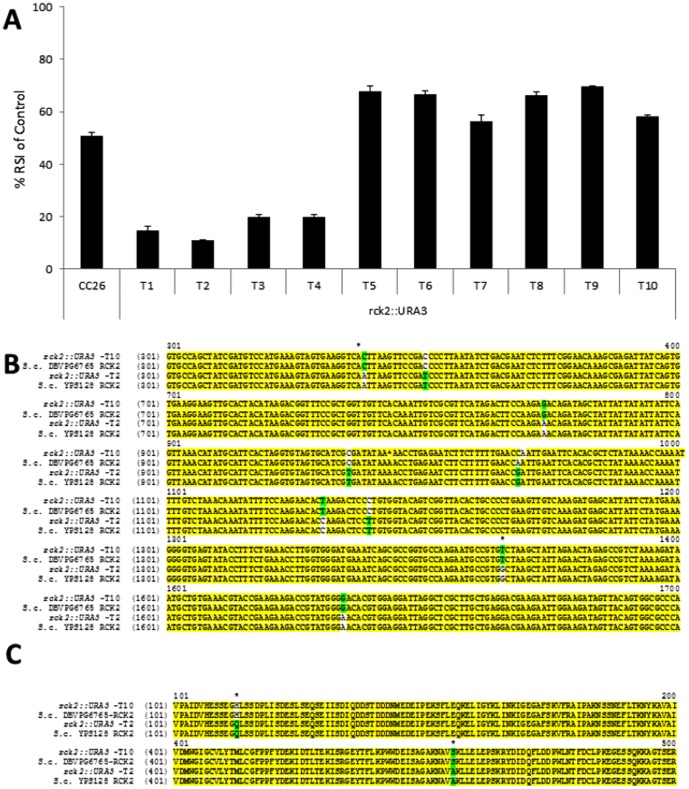
Phenotypic microarray screening of (A) heterozygous diploid transformants (transformants labelled T1–T10) to osmotic stress harbouring reciprocal deletions of *RCK2* alleles (B) DNA and (C) Protein sequence comparison of *RCK2*-*::rck2* T2 and T10 transformants and their parental strains DBVPG6567 and YPS128.

We examined the genes present within the QTL identified under osmotic stress on chromosome XII and selected *HSP60*, *RCK2*, *GSY1* and *PUT1* as candidate genes for reciprocal hemizygosity analysis. We observed using the phenotypic microarray screen that heterozygous diploid transformants harbouring different *RCK2* or *HSP60* alleles exhibited different tolerances to osmotic stress (Figure 6A). Sequencing the wild-type *HSP60* and *RCK2* alleles in these diploid heterozygous strains revealed nucleotide and peptide differences for *RCK2*, however, we failed to discern any differences in nucleotide or peptide sequences for *HSP60*. Tolerant transformants carried the *RCK2* allele inherited from strain DBVPG6765 (Figure 6B) which has been previously shown to display tolerance to osmotic stress when compared with other *Saccharomyces spp* strains [24].

*RCK2* from DBVPG6765 has a glutamic acid at residue 113 and a serine at residue 456, while *RCK2* from YPS128 has a histidine at residue 113 and an alanine at residue 456, respectively (Figure 6C). Sequence analysis revealed that all *S. paradoxus* strains and 56% (22/39) of *S. cerevisiae* strains contained a glutamic acid at residue 113, and an alanine at residue 456. This included the yeast reference strain S288c. Approximately 39% (15/39) of the *S. cerevisiae* strains in the SGRP collection contained a histidine at residue 113 and a serine at residue 456, respectively (data S8). Two *S. cerevisiae* strains had a histidine at residue 113 but had a serine at residue 456 (data S8). These yeast have previously been identified as having a mosaic genome [10]. Heterozygous diploid transformants harbouring deletions of *PUT1* and GSY2 did not exhibit any changes in their tolerance to osmotic stress when compared to their isogenic parents; this was confirmed for both alleles using sequencing.

## Discussion

A robust yeast strain tolerant to all inhibitory conditions and pre-treatment inhibitors exposed to during bioethanol fermentation has yet to be identified. In this study, we performed linkage analysis using divergent *S. cerevisiae* clean lineages to map bioethanol relevant QTL's. We analysed F1 segregants for their response to stresses present in bioethanol fermentation using a phenotypic microarray assay and observed F1 haploid segregants derived from six pairwise crosses of *S. cerevisiae* clean lineages are as being phenotypically distinct to either parent. This observation agrees with previous studies that phenotypic variation can be displayed in progeny from F1 hybrids when compared with parental strains, including increased vigour. Transgressive variation for stress tolerance has been described previously for heat and oenological phenotypes in haploid yeast strains but not for fermentation stresses [17].

Mapping QTL's to a phenotype in yeast has been successful for desired traits such as ethanol tolerance [25], sensitivity to heavy metals or pesticides [26] and performance of yeast in a fermentation [27], however, QTL's desirable for bioethanol fermentations have not been published previously. A QTL identifying an asparaginase from wine yeast haploid segregants producing acetic acid was identified, however, this QTL was only apparent when yeast were utilising asparagine as the sole nitrogen source [28]. We identified QTL's related to weak acid stress and to osmotic stress, within the QTL's we identified genes whose expression changed under stress conditions.

We performed reciprocal hemizygosity analysis of candidate genes within each QTL, demonstrating that an allele of *COX20*, a mitochondrial cytochrome C oxidase gene conferred acetic acid tolerance. This phenotype was dependent on which parental allele had been inherited and sensitive progeny contained *COX20* from strain Y12. This strain has been previously identified as being sensitive to acetic acid in comparison to DBVPG6044 [24]. DNA sequence analysis of *COX20* revealed that the acetic acid tolerant yeast strain (DBVPG6044) has a glutamic acid at residue 9 whereas the acetic acid sensitive strain (Y12) has an arginine residue at this position.

Cytochrome C oxidase activity has been associated with programmed cell death (PCD) in yeast [29], where a loss of function along with addition of acetic acid has been shown to induce PCD [30]. Yeast strains with altered cytochrome C oxidase activity maybe more tolerant to the inducement of PCD by acetic acid, the importance of cytochrome C oxidase has been reported in work on improving acetate tolerance in *E. coli* [31].

Applying reciprocal hemizygosity to candidate genes within the QTL identified under osmotic stress, null alleles of rck2 and hsp60 were generated in the YPS128×DBVPG6567 F1 hybrid. It was demonstrated that *RCK2* mediated osmotic tolerance was dependent on the inherited parental allele. Sensitive heterozygous diploid transformants contained the *RCK2* allele from the parental strain YPS128 and resistant progeny from DBVPG6567. *RCK2* is a protein kinase which has a known regulatory role in the Hog1 pathway [32] and has been previously highlighted for response to oxidative and osmotic stress in yeast, particularly salt tolerance [33], [34]. QTL analysis has worked in plant cell lines under osmotic stress highlighting variations between different cultivars of *Arabidopsis* [35] and wheat [36] and identifying loci on chromosomes specifically for plant response under osmotic stress.

Expression data revealed that HSP60 was significantly up-regulated under osmotic stress, furthermore, differential response levels were observed among *HSP60::hsp60* transformants under osmotic stress. Heat shock proteins have been observed to play key roles in response to other stress conditions in *S. cerevisiae* such as freezing, oxidative and temperature stress [37], [38]. *HSP60* has been identified as a novel target site to understand the direct relationship between osmotic and heat shock stress response to find novel (QTLs) target sites for strain improvement. Surprisingly we did not find any protein sequence differences between these alleles; therefore we haven't identified a rationale for the phenotypic variation, alignment studies of HSP60 in *Saccharomyces* spp has revealed that this protein is highly conserved with minimal variation in amino acids across the genus (data not shown) indicating that differences in expression of the gene between the two alleles rather than sequence variation could be responsible for the variation observed in the transformants, this is currently being pursued.

Analysis of other candidate genes such as *ADH3*, *GCN2*, *MSN2*, and *AAC1* (acetic acid) and *PUT1*, *GSY1* (osmotic stress) exhibited no differences between transformants (PCR analysis revealed that some of each allele had been knocked out) to the relevant stress even under stress levels greater than originally used in the phenotypic screen.

We have looked at some of the candidate genes within the QTL's, however, we haven't analysed all the genes within the QTL's so other candidate genes responsible for the tolerance to stress could be present. Despite extensive experiments we were unable to identify QTL's for other stresses inherent to bioethanol fermentations such as HMF, furfural, vanillin, ethanol, or increasing temperature despite phenotypic variation between the segregants. QTL analysis is not without limitations such as the requirements for large sample size and can only map differences inherent in the parental strains [39] so QTL's for these traits maybe present in other haploid yeast populations as the sample size was too small and the linkage disequilbrium (LD) was too big as it is only a one generation cross, ethanol tolerance in larger populations has been successful in identifying transcription factors influencing yeast phenotypes [25], [40].

There were other genes chosen for reciprocal hemizygosity analysis but no difference in phenotypes between alleles was observed. We acknowledge that not all genes within all loci were examined in this study by reciprocal hemizygosity analysis and that additional genes within these loci may also contribute to resistance of fermentation inhibitors within *S. cerevisiae* strains. As we were working with F1 segregant populations in this study with limited crossing-over events, the QTL's that we identified contained between 40 and 60 genes due to large blocks of linked SNPs. Further crosses between the F1 segregants used in this study and crosses of subsequent populations derived from them will enable us to shorten the LD blocks and eventually facilitate the identification of loci contributing to a trait at a single gene level as has been done for heat tolerance [41].

In conclusion, our studies have revealed QTL's from yeast haploid populations under stress and has highlighted allelic variation (*COX20* or *RCK2*) and changes in gene expression levels (*HSP60* and *COX20*) under stress conditions. This study has highlighted the phenotypic variation for any population of yeast to stresses inherent to bio-ethanol fermentations, using this approach we have identified chromosomal regions responsible for the genetic and molecular basis for natural variation in bioethanol traits.

## Supporting Information

Figure S1 **Phenotypic microarray analysis (redox signal intensity) of F1 haploid segregants for tolerance to (A) 25 mM acetic acid (B) 10 mM formic acid, (C) 10 mM furfural (D) 10 mM HMF, (E) 10 mM vanillin, (F) 20% sorbitol, (G) 5 10% ethanol, (H) 35°C are shown.** Slide 1 – Data from F1 haploid segregants from *S. cerevisiae* DBVPG6765 and YPS128, slide 2 - Data from F1 haploid segregants from *S. cerevisiae* DBVPG6765 and Y12, slide 3 - Data from F1 haploid segregants from *S. cerevisiae* DBVPG6765 and DBVPG6044, slide 4 - Data from F1 haploid segregants from *S. cerevisiae* YPS128 and DBVPG6044, slide 5 - Data from F1 haploid segregants from *S. cerevisiae* YPS128 and Y12, and slide 6 - Data from F1 haploid segregants from *S. cerevisiae* DBVPG6044 and Y12. The values shown are an average of triplicate experiments including standard deviations.
(PPTX)

Data S1 **Phenotypic microarray data for F1 haploid segregants from** ***S. cerevisiae*** **YPS128 and Y12.**
(XLSX)

Data S2 **Phenotypic microarray data for F1 haploid segregants from** ***S. cerevisiae*** **YPS128 and DBVPG6044.**
(XLSX)

Data S3 **Phenotypic microarray data for F1 haploid segregants from** ***S. cerevisiae*** **DBVPG6765 and Y12.**
(XLSX)

Data S4 **Phenotypic microarray data for F1 haploid segregants from** ***S. cerevisiae*** **DBVPG6765 and Y12.**
(XLSX)

Data S5 **Phenotypic microarray data for F1 haploid segregants from** ***S. cerevisiae*** **DBVPG6044 and Y12.**
(XLSX)

Data S6 **Phenotypic microarray data for F1 haploid segregants from** ***S. cerevisiae*** **DBVPG6044 and DBVPG6765.**
(XLSX)

Data S7 **Loci identified by QTL for relevant stress, source of F1 haploid cell lines, and tabulated gene functions from within the relevant loci.**
(XLSX)

Data S8 **Peptide alignment for RCK2 (** **tab 1** **) using peptide sequences form** ***S. cerevisiae*** **and** ***S. paradoxus*** **strains, peptide alignment for COX20 (** **tab 2** **) using peptide sequences form** ***S. cerevisiae*** **and** ***S. paradoxus*** **strains.**
(XLSX)
