# Correction: The Genetic Basis of Variation in Clean Lineages of *Saccharomyces cerevisiae* in Response to Stresses Encountered during Bioethanol Fermentations

**DOI:** 10.1371/journal.pone.0119343

**Published:** 2015-03-27

**Authors:** The PLOS ONE Staff

There are a number of errors in the legend for Fig. 1, “Phenotypic microarray analysis (redox signal intensity) of F1 haploid segregants from a Y12×DBVPG6044 cross.” The complete, correct Fig. 1 legend is:

**Fig 1:**
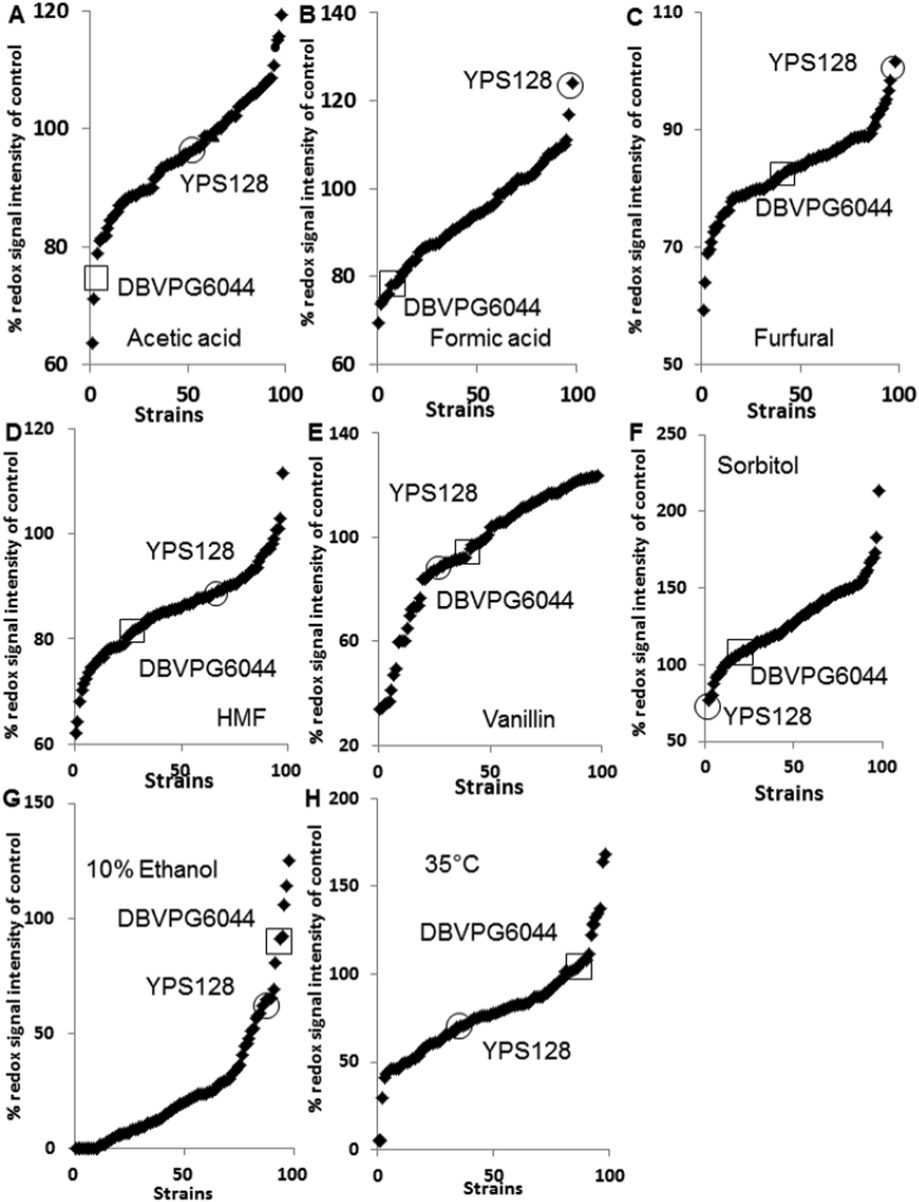
Phenotypic microarray analysis (redox signal intensity) of F1 haploid segregants from a Y128×DBVPG6044 cross. Tolerance to (A) 25 mM acetic acid (B) 10 mM formic acid, (C) 10 mM furfural (D) 10 mM HMF, (E) 10 mM vanillin, (F) 20% sorbitol, (G) 10% ethanol, (H) 35°C are shown. The Y axis represents the % of RSI (redox signal intensity) where wells containing the listed stresses are compared to unstressed conditions. All yeast cells were grown in minimal medium with 6% glucose added at 30°C with the final data shown at the 25 hr time point. The values shown are an average of triplicate experiments including standard deviations.
