# Supplementary material for: The Genetic Basis of Variation in Clean Lineages of Saccharomyces cerevisiae in Response to Stresses Encountered during Bioethanol Fermentations
Source: PLoS One. 2014 Aug 12;9(8):e103233. doi: 10.1371/journal.pone.0103233 (PMC4130530; doi:10.1371/journal.pone.0103233)
Supplement: Figure S1 — Phenotypic microarray analysis (redox signal intensity) of F1 haploid segregants for tolerance to (A) 25 mM acetic acid (B) 10 mM formic acid, (C) 10 mM furfural (D) 10 mM HMF, (E) 10 mM vanillin, (F) 20% sorbitol, (G) 5 10% ethanol, (H) 35°C are shown. Slide 1 – Data from F1 haploid segregants from S. cerevisiae DBVPG6765 and YPS128, slide 2 - Data from F1 haploid segregants from S. cerevisiae DBVPG6765 and Y12, slide 3 - Data from F1 haploid segregants from S. cerevisiae DBVPG6765 and DBVPG6044, slide 4 - Data from F1 haploid segregants from S. cerevisiae YPS128 and DBVPG6044, slide 5 - Data from F1 haploid segregants from S. cerevisiae YPS128 and Y12, and slide 6 - Data from F1 haploid segregants from S. cerevisiae DBVPG6044 and Y12. The values shown are an average of triplicate experiments including standard deviations. (PPTX) [file pone.0103233.s001.pptx]

## Slide 1
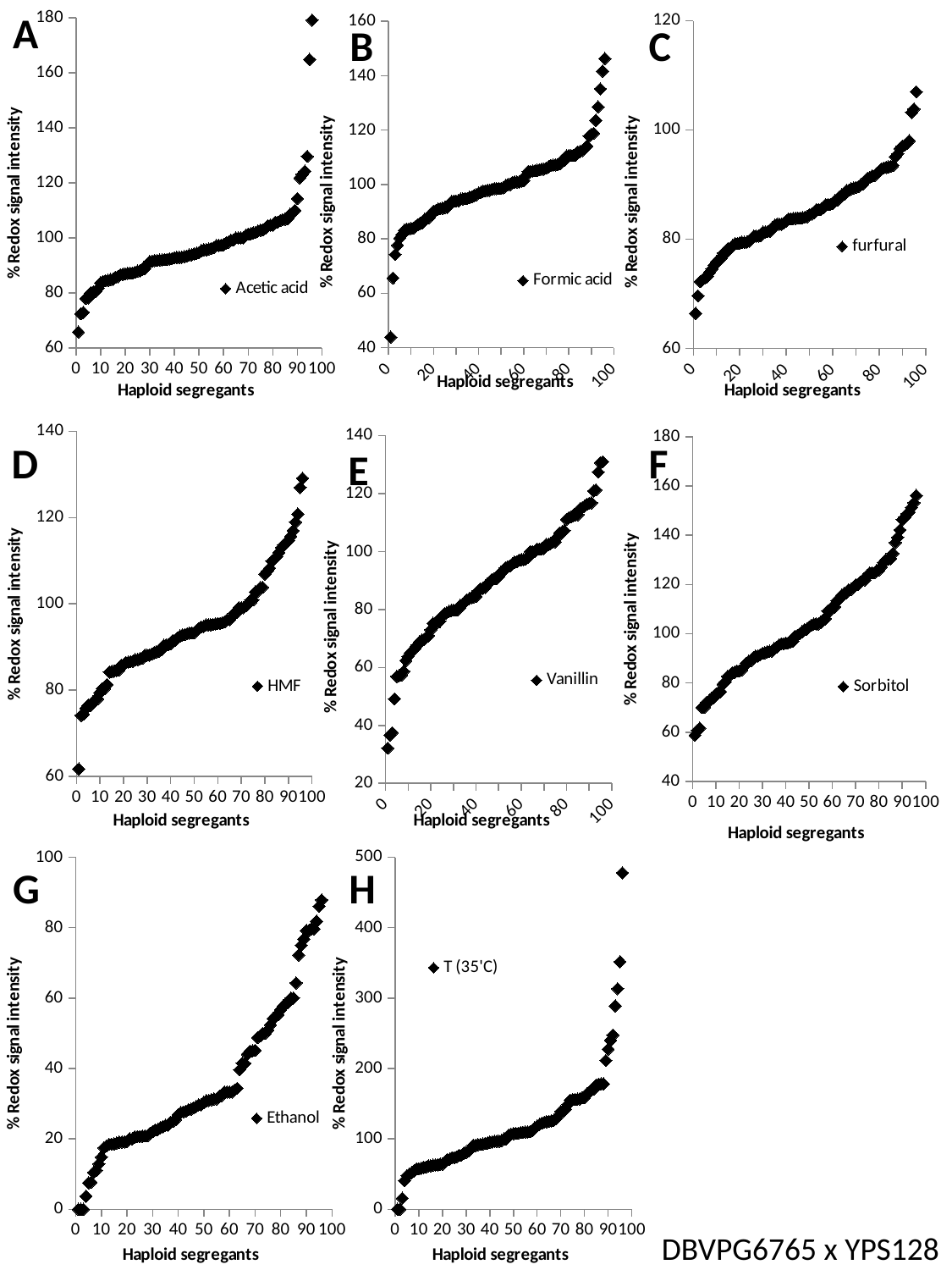

A
### Chart
| Category | furfural |
|---|---|
### Chart
| Category | Acetic acid |
|---|---|
### Chart
| Category | Formic acid |
|---|---|B
C
### Chart
| Category | HMF |
|---|---|
### Chart
| Category | Sorbitol |
|---|---|
### Chart
| Category | Vanillin |
|---|---|F
D
E
### Chart
| Category | Ethanol |
|---|---|
### Chart
| Category | T (35'C) |
|---|---|G
H
DBVPG6765 x YPS128

## Slide 2
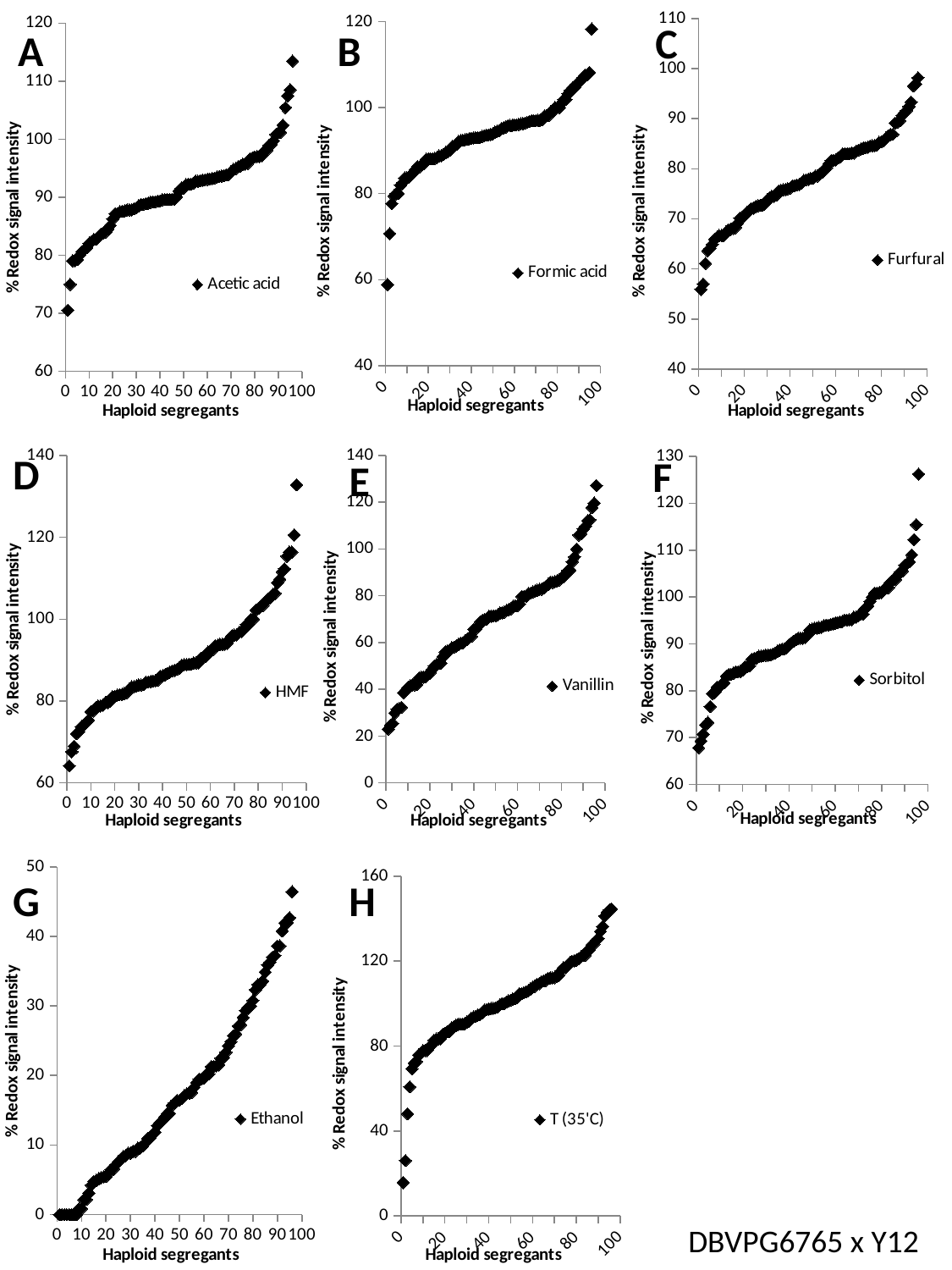

### Chart
| Category | Formic acid |
|---|---|
### Chart
| Category | Furfural |
|---|---|
### Chart
| Category | Acetic acid |
|---|---|C
A
B
### Chart
| Category | HMF |
|---|---|
### Chart
| Category | Vanillin |
|---|---|
### Chart
| Category | Sorbitol |
|---|---|D
F
E
### Chart
| Category | Ethanol |
|---|---|
### Chart
| Category | T (35'C) |
|---|---|G
H
DBVPG6765 x Y12

## Slide 3
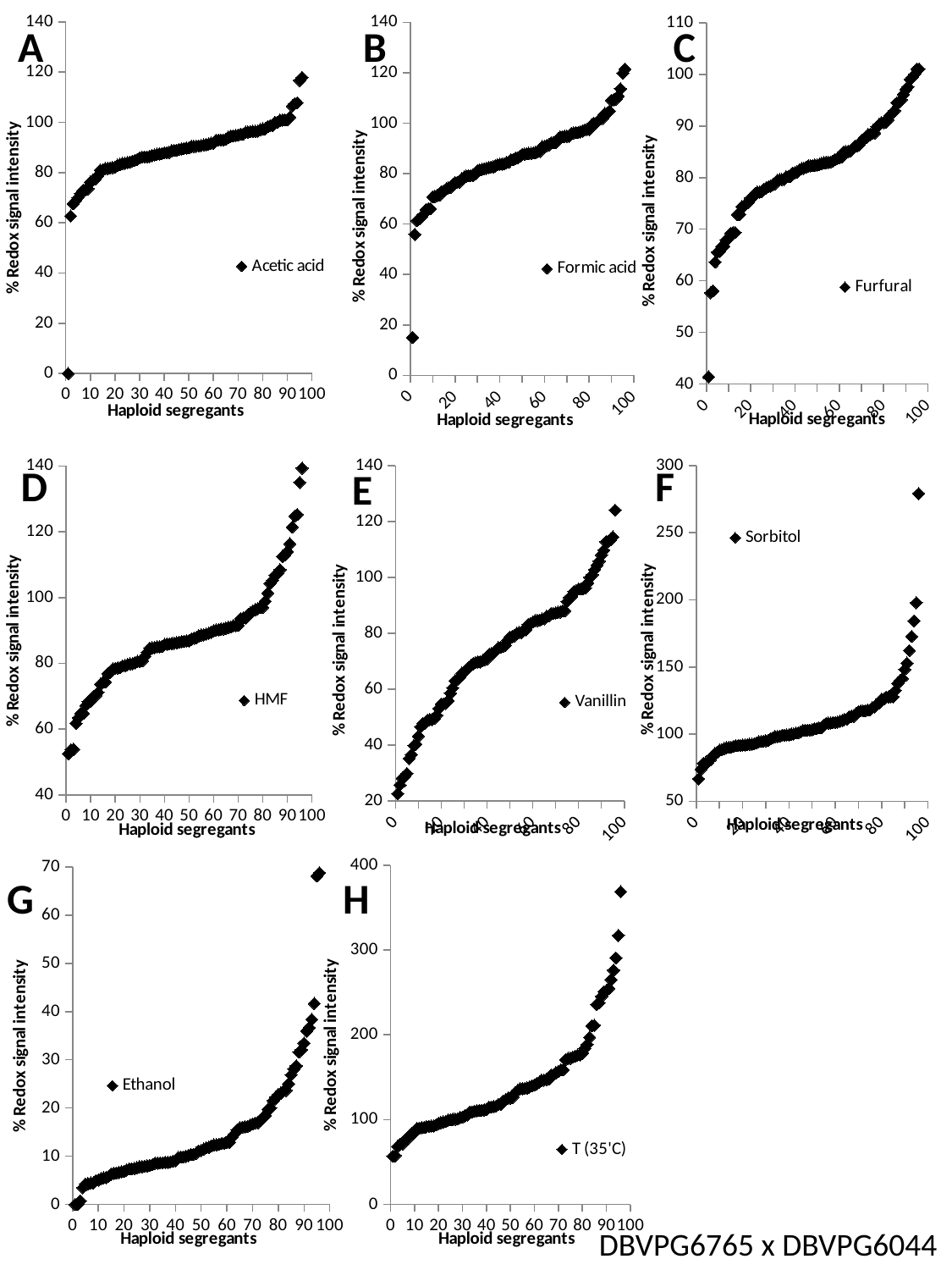

### Chart
| Category | Acetic acid |
|---|---|
### Chart
| Category | Formic acid |
|---|---|
### Chart
| Category | Furfural |
|---|---|C
A
B
### Chart
| Category | HMF |
|---|---|D
F
### Chart
| Category | Vanillin |
|---|---|
### Chart
| Category | Sorbitol |
|---|---|E
### Chart
| Category | T (35'C) |
|---|---|
### Chart
| Category | Ethanol |
|---|---|G
H
DBVPG6765 x DBVPG6044

## Slide 4
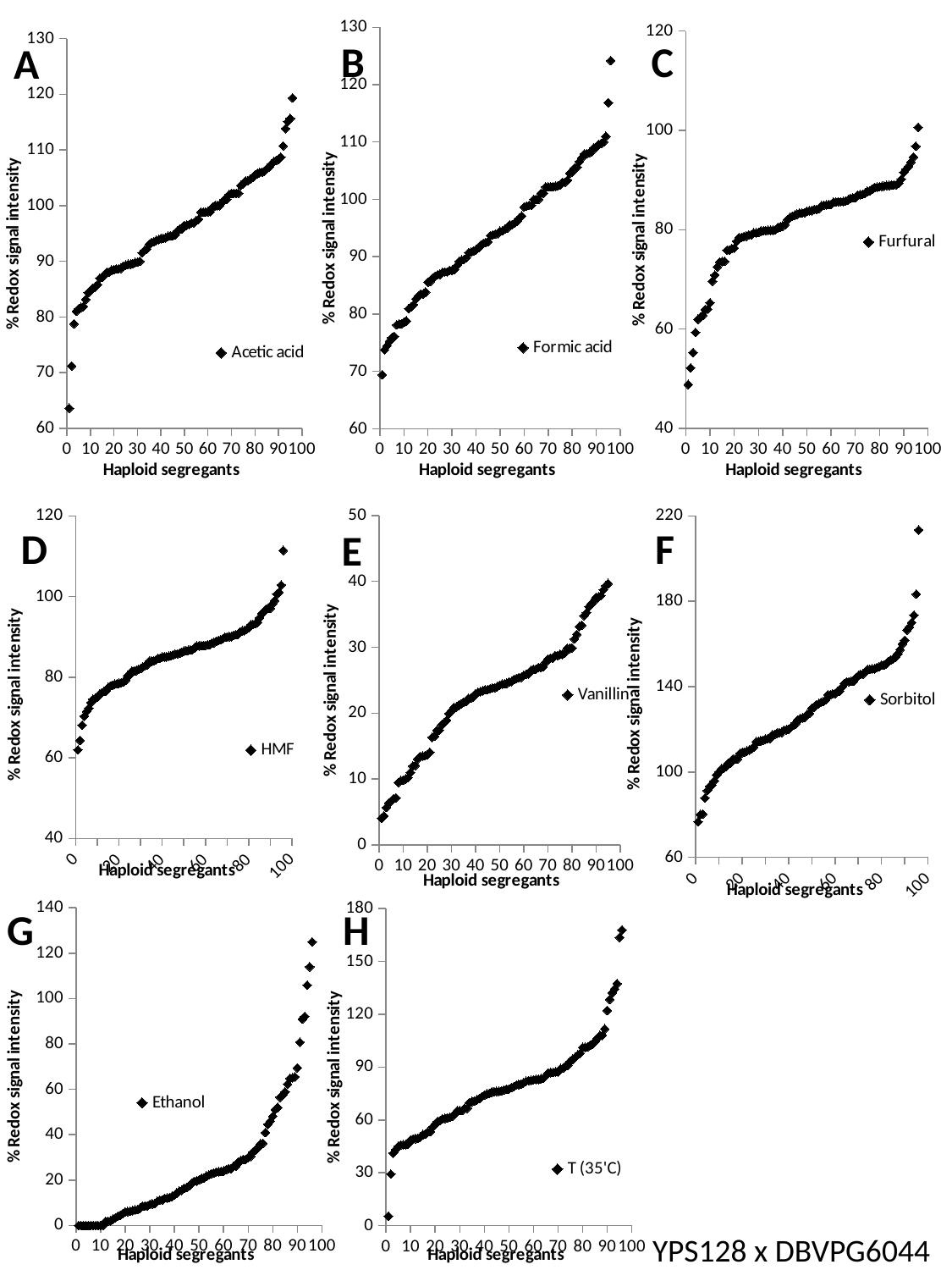

### Chart
| Category | Furfural |
|---|---|
### Chart
| Category | Acetic acid |
|---|---|
### Chart
| Category | Formic acid |
|---|---|C
B
A
### Chart
| Category | HMF |
|---|---|
### Chart
| Category | Vanillin |
|---|---|
### Chart
| Category | Sorbitol |
|---|---|D
F
E
### Chart
| Category | Ethanol |
|---|---|
### Chart
| Category | T (35'C) |
|---|---|G
H
YPS128 x DBVPG6044

## Slide 5
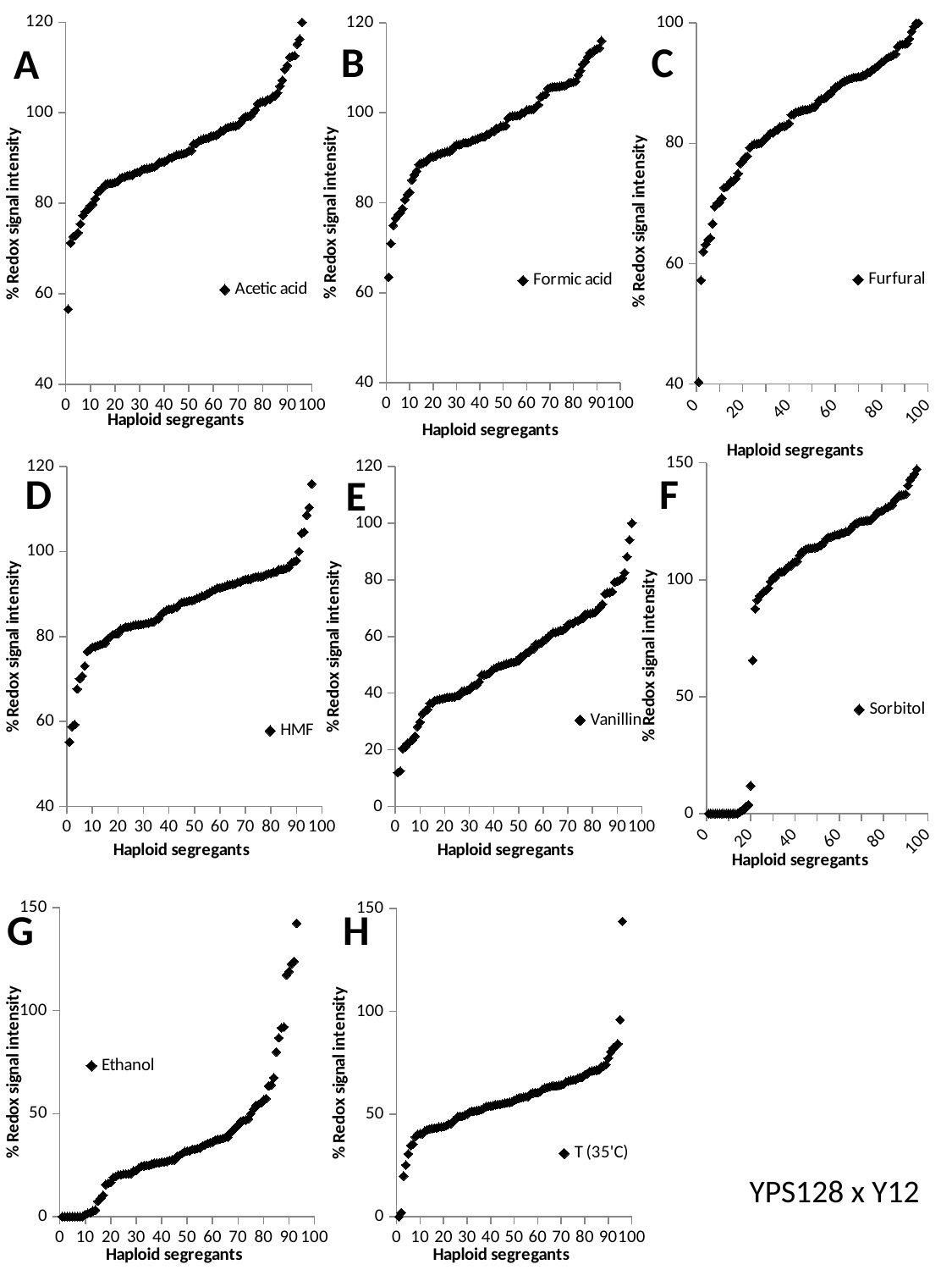

### Chart
| Category | Acetic acid |
|---|---|
### Chart
| Category | Formic acid |
|---|---|
### Chart
| Category | Furfural |
|---|---|C
B
A
### Chart
| Category | Sorbitol |
|---|---|
### Chart
| Category | HMF |
|---|---|
### Chart
| Category | Vanillin |
|---|---|D
F
E
### Chart
| Category | Ethanol |
|---|---|
### Chart
| Category | T (35'C) |
|---|---|G
H
YPS128 x Y12

## Slide 6
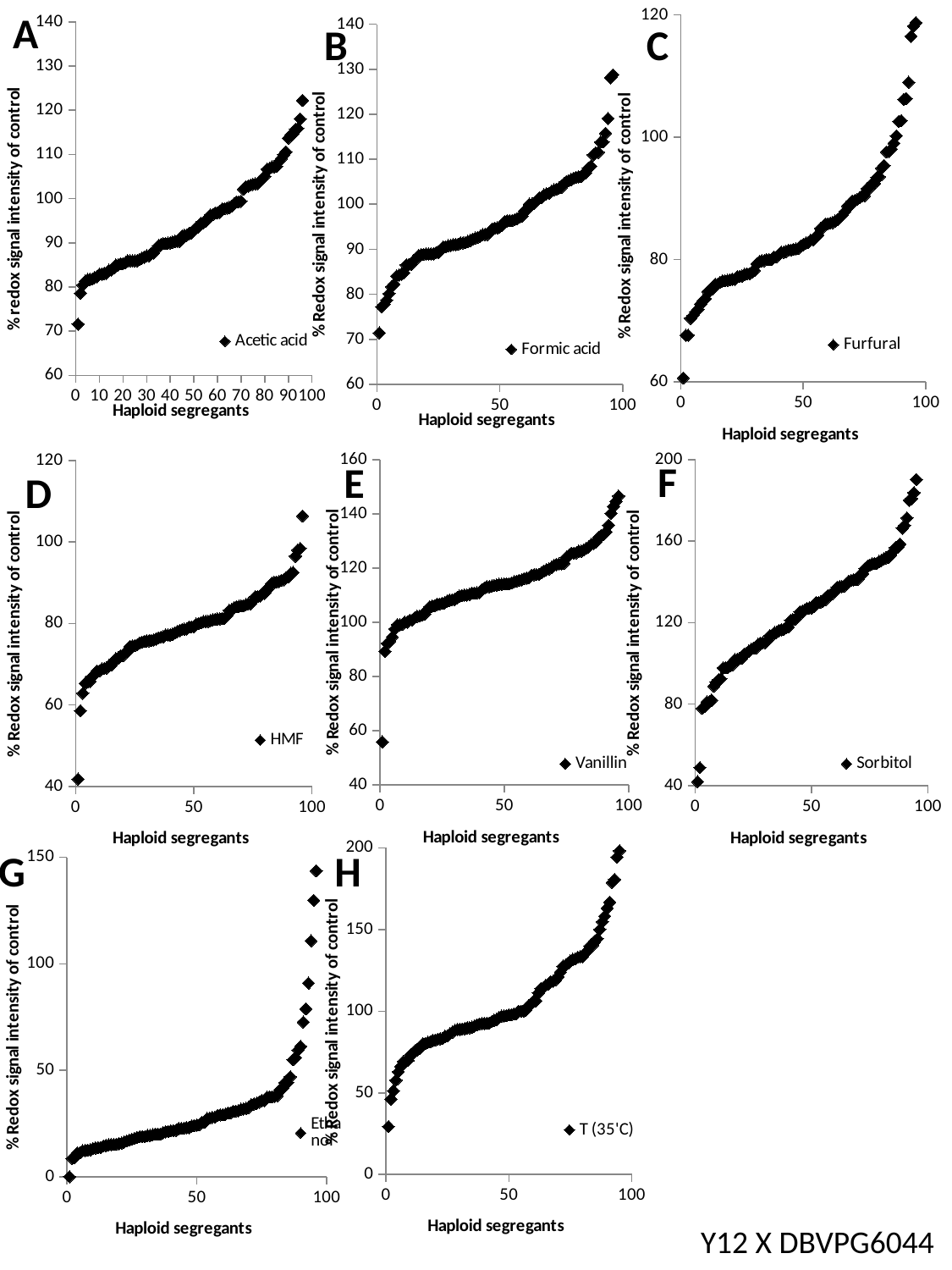

### Chart
| Category | Acetic acid |
|---|---|A
### Chart
| Category | Formic acid |
|---|---|
### Chart
| Category | Furfural |
|---|---|C
B
### Chart
| Category | HMF |
|---|---|
### Chart
| Category | Vanillin |
|---|---|
### Chart
| Category | Sorbitol |
|---|---|F
E
D
### Chart
| Category | T (35'C) |
|---|---|
### Chart
| Category | Ethanol |
|---|---|G
H
Y12 X DBVPG6044
